# Linking drug and food addiction: an overview of the shared neural circuits and behavioral phenotype

**DOI:** 10.3389/fnbeh.2023.1240748

**Published:** 2023-09-12

**Authors:** Alice Passeri, Diana Municchi, Giulia Cavalieri, Lucy Babicola, Rossella Ventura, Matteo Di Segni

**Affiliations:** ^1^IRCCS Fondazione Santa Lucia, Rome, Italy; ^2^Department of Psychology and Center “Daniel Bovet”, Sapienza University, Rome, Italy; ^3^IRCCS San Raffaele, Rome, Italy

**Keywords:** animal models, eating disorders, food addiction, substance use disorder, eating addiction

## Abstract

Despite a lack of agreement on its definition and inclusion as a specific diagnosable disturbance, the food addiction construct is supported by several neurobiological and behavioral clinical and preclinical findings. Recognizing food addiction is critical to understanding how and why it manifests. In this overview, we focused on those as follows: 1. the hyperpalatable food effects in food addiction development; 2. specific brain regions involved in both food and drug addiction; and 3. animal models highlighting commonalities between substance use disorders and food addiction. Although results collected through animal studies emerged from protocols differing in several ways, they clearly highlight commonalities in behavioral manifestations and neurobiological alterations between substance use disorders and food addiction characteristics. To develop improved food addiction models, this heterogeneity should be acknowledged and embraced so that research can systematically investigate the role of specific variables in the development of the different behavioral features of addiction-like behavior in preclinical models.

## 1. Introduction

Eating is a multifaceted behavior determined by physiological and motivational drives and modulated by several mechanisms that include environmental, emotional, social, and cultural factors (Emilien and Hollis, 2017). Eating behavior can also lead to disabling conditions such as eating disorders (EDs), which are characterized by complex biological and environmental interactions (Bulik et al., 2022). Recently, clinical and preclinical studies have focused on the environmental risk factors for the development of dysfunctional eating behaviors and, specifically, on the nutrient composition of high-calorie, high-fat, high-sugar, and highly processed foods (Gibney et al., 2017; Monteiro et al., 2019; Hecht et al., 2022; Wiss, 2022).

These foods, alternatively referred to as hyperpalatable, highly processed, and highly rewarding foods (HPFs), can trigger those brain areas controlling gratification processing (liking) and attribution of salience (wanting), as proposed by the incentive sensitization theory, developed as a framework for SUDs’ interpretation (Berridge et al., 2010; Gearhardt et al., 2011; Volkow et al., 2012; Schulte et al., 2015; Berridge and Robinson, 2016; Robinson et al., 2016; Cameron et al., 2017; Devoto et al., 2018; Lindgren et al., 2018; Volkow et al., 2019; Stice and Yokum, 2021). According to the recent NOVA classification (Monteiro et al., 2010), even if ultra-processed foods and drinks (UPFDs) provide calories, they are poor sources of micronutrients and possess low satiating power (Monteiro et al., 2018). In addition, increased consumption of these foods is strongly associated with obesity, diet-related diseases, and adverse mental health symptoms (Gearhardt and Schulte, 2021; Hecht et al., 2022).

There is a consensual definition of UPFDs as highly palatable and gratifying foods, whose organoleptic features can affect physiological mechanisms involved in satiety and appetite regulation, which may promote excessive consumption and manifest as a true “addiction” (Gibney et al., 2017). Food addiction (FA) (Randoph, 1956) has been described in a range of terms including eating addiction (Hebebrand et al., 2014), sugar addiction (Avena et al., 2008), fat addiction (Sarkar et al., 2019), HPF addiction (Gearhardt et al., 2011), salted FA (Cocores and Gold, 2009), and, recently, UPFDs addiction (Wiss, 2022). Disagreement upon the terminology belies a debate concerning the validity of the FA construct and its mechanisms (Ziauddeen et al., 2012; Salamone and Correa, 2013; Ziauddeen and Fletcher, 2013; Hebebrand et al., 2014; Westwater et al., 2016; Finlayson, 2017; Rogers, 2017; Fletcher and Kenny, 2018; Lacroix et al., 2018; Gearhardt and Hebebrand, 2021a).

Researchers have argued that there is a strong overlap between clinical features of FA and binge eating disorder (BED) and that human studies assessing neurobiological mechanisms of FA are not convincing. In addition, animal models of FA seem to lack ecological validity (Fletcher and Kenny, 2018). Therefore, some authors support the behavioral addiction hypothesis (eating addiction) based on the argument that substances of abuse act as agonists of specific brain receptors and have a direct effect on the reward system. This is not the case for food. Moreover, eating is necessary for survival and intrinsically rewarding (Hebebrand et al., 2014; Gearhardt and Hebebrand, 2021a). However, other authors support the FA hypothesis, emphasizing behavioral and neurobiological similarities between HPF consumption and substance use disorders (SUDs) (Meule and Gearhardt, 2014; Gordon et al., 2018; Wiss et al., 2018; Gearhardt and Schulte, 2021). They strongly support FA as an independent clinical entity characterized by a specific behavioral pattern, including compulsive overeating [(Davis, 2017), for extensive reviews, see, Gearhardt and Hebebrand (2021a,b) and Hebebrand and Gearhardt (2021)]. Nevertheless, it has been proposed that the psychological constructs related to overeating (e.g., binge eating, food addiction, food craving, and hedonic hunger) share phenotypic, genetic, and environmental features that could be enclosed under the umbrella term “uncontrolled eating,” conceptualized as high food reward sensitivity combined with poor self-control, thus distancing it from the addiction hypothesis (Vainik et al., 2019, 2020). According to the DSM-5-TR (American Psychiatric Association, 2022), the diagnosis of SUD is based on pathological behaviors related to substance use. It identifies different forms of substance-related and addictive disorders and “non-substance-related addictions,” which refer only to gambling disorders. Even if FA is not recognized as a diagnosable pathology, its clinical features—especially loss of control and craving, risky use, tolerance, and withdrawal—seem to fit with the diagnostic criteria for SUDs. Indeed, these criteria have been used for the development and validation of tools to assess FA in humans, including the “Yale Food Addiction Scale,” the “Three Factors Eating Questionnaire,” the “Power of Food Scale,” and the “Loss of Control over Eating Scale” (Stunkard and Messick, 1985; Lowe et al., 2009; Gearhardt et al., 2011, 2016; Latner et al., 2014; Pursey et al., 2015; Schulte et al., 2015; de Vries and Meule, 2016; Burrows et al., 2017; Markus et al., 2017; Ayaz et al., 2018; Lemeshow et al., 2018; Carter et al., 2019; Tran et al., 2020; Sanchez et al., 2022). Interestingly, both clinical and preclinical studies have suggested how the mesolimbic dopamine (DA) system, mediating reward-related stimuli, learning, motivational, and executive control processes (Wang et al., 2004; Stice et al., 2008; Belin et al., 2009; Wise, 2009; Volkow et al., 2012; Tomasi and Volkow, 2013; Furlong et al., 2014; Volkow et al., 2017; Novelle and Diéguez, 2018; Volkow et al., 2019; Ndiaye et al., 2020) is involved both in SUDs and FA. Repeated stimulation of the DA-reward pathway is believed to foster the sensitization of the mesolimbic system both to the substance itself and its associated cues (Berridge and Robinson, 2016) and to promote neurobiological adaptations that result in the development of pathological behaviors such as binging (Furlong et al., 2014), craving (Krasnova et al., 2014; Madangopal et al., 2022), use despite negative consequences (Latagliata et al., 2010), withdrawal, and tolerance (Avena et al., 2005, 2008; Iemolo et al., 2012; Sharma et al., 2013).

## 2. The mesoaccumbens dopamine system involvement in drug and food addiction

HPF and drugs of abuse can impact the function of similar brain circuits, including the mesoaccumbens DA system (Latagliata et al., 2010; Volkow et al., 2017; Fletcher and Kenny, 2018). It is worth pointing out how the “reward” system responds to both rewarding and aversive motivational stimuli (Salamone and Correa, 2012). Therefore, alteration of this system could result in various psychopathologies characterized by impaired motivational stimuli processing, such as depression, schizophrenia, Parkinson’s disease, and addiction-like disorders (Salamone et al., 2016). Interestingly, it seems that both natural rewards and drugs of abuse exert initial reinforcing effects by targeting brain regions of this circuit, such as the nucleus accumbens (NAc). However, addiction results from the transition from hedonic intake to an uncontrolled one, which requires long-lasting adaptations within the reward system and associated circuits (Domingo-Rodriguez et al., 2020).

### 2.1. The prefrontal cortex

The prefrontal cortex (PFC) is involved in the regulation of cognitive flexibility, decision-making, and inhibitory control. It plays a crucial role in the transition to and persistence of addictive behavior (Domingo-Rodriguez et al., 2020). An impairment in executive functions is likely to contribute to the poor control and high compulsivity seen in addicted subjects (Volkow and Baler, 2014; Volkow et al., 2019).

PFC regions are activated when cocaine abusers are exposed to craving-inducing stimuli, and the increase in metabolic activity in the orbitofrontal cortex (OFC) and anterior cingulate cortex (ACC) is associated with the intensity of the craving (Volkow and Baler, 2014; Volkow et al., 2019). Neuroimaging studies show inhibition of metabolic activity in OFC (Volkow and Baler, 2014; Koob and Volkow, 2016) and ACC (Koob and Volkow, 2016) in cocaine abusers when they are asked to inhibit craving after exposure to cocaine cues (Volkow and Baler, 2014; Koob and Volkow, 2016).

Interestingly, a similar metabolic inhibition can be observed in obese patients when they are asked to inhibit craving for food after they have been exposed to food cues (Volkow and Baler, 2014). In human studies, subjects with BED and bulimia nervosa show weakened functional connectivity between the left lateral OFC, which is implicated in the inhibitory suppression of rewarded choices (Mar et al., 2011; Gourley et al., 2016) and the right dorsolateral PFC (DLPFC) (Ahn et al., 2022), which mediates the relationship between motor urgency and response inhibition (Hu et al., 2016). When exposed to high-calorie food stimuli, individuals with BED display an overactivation of the OFC (Schienle et al., 2009; Hone-Blanchet and Fecteau, 2014; Meng et al., 2020; Celeghin et al., 2023). Notably, lateral OFC is crucial for goal-directed behavior. A recent study showed how in a mouse model of obesity, lateral OFC-dependent impairments in devaluation (mediated by GABAergic transmission) may alter the ability to use the value of the outcome to guide behavior (Seabrook et al., 2023). Increased activation of OFC is also evident in drug-addicted subjects in response to drug-related cues (Sell et al., 2000; Wang et al., 2007; Kufahl et al., 2008; Goldstein and Volkow, 2011; Ceceli et al., 2022), and it is considered an index of craving (Volkow et al., 2010).

Food- and drug-addicted subjects demonstrate executive function impairment during recovery and relapse (Basso et al., 2022), similar brain activation during craving (Gearhardt et al., 2011) with specific and predictive neuromarkers (“neurobiological craving signature”) (Koban et al., 2023), and the transition from goal-directed action to habits (DiFeliceantonio et al., 2018). Furthermore, the modulation of craving and consumption of alcohol, nicotine, drugs, or food by excitatory neuromodulation interventions of DLPFC has been suggested (Song et al., 2022).

Preclinical studies have also shown similar cortical alterations between drug abuse and FA (Chen et al., 2013; Limpens et al., 2015; Newmyer et al., 2019; Domingo-Rodriguez et al., 2020; Amissah et al., 2021; Navandar et al., 2021). In addition, vulnerability to drug addiction and FA shares several transcriptional signatures in mPFC (Navandar et al., 2021). The activity of the prelimbic cortex, implicated in response inhibition (Chen et al., 2013; Limpens et al., 2015), seems to correlate with compulsive seeking of both drugs and food (Chen et al., 2013; Domingo-Rodriguez et al., 2020). Furthermore, the medial insula plays a critical role in cravings for food, cocaine, and nicotine (Mar et al., 2011; Volkow and Baler, 2014). Insular reactivity has been proposed as a potential biomarker for relapse risk and a target for addiction treatments (Volkow et al., 2019). Imaging studies have reported differential activation of the insula during craving, possibly reflecting interoceptive cues and the activation of corticotropin-releasing factor (Mar et al., 2011). The OFC-anterior insular cortex pathway is also involved in cocaine addiction (Chen et al., 2022) and, interestingly, using a continuous versus intermittent cocaine self-administration paradigm, it has been found that the latter was able to induce greater cocaine-taking behavior during withdrawal (Luo et al., 2021). The same effect, mediated by an intermittent (but not continuous) self-administration paradigm, has been reported for HPF (Spierling et al., 2020).

### 2.2. The nucleus accumbens

The NAc is a central area of the reward circuit and an important driver of goal-directed and goal-associated actions (Volkow et al., 2019). It is involved in several addiction-related processes, such as memory, learning, and response inhibition (Volkow and Baler, 2014). One of the changes related to the increased responsiveness to drug-predictive cues in addiction is the balance between D1 and D2 receptors (D1R, D2R) signaling in the ventral striatum. Preclinical studies support the idea that strengthening of D1R-medium spiny neurons (MSNs) in NAc enhances cocaine reward, whereas strengthening of D2R-MSNs suppresses it (Volkow et al., 2019). Similarly, mice fed with a high-fat diet show enhanced activity of NAc D1R-MSNs during food seeking, which is linked to increased excitatory synaptic drive in the same neurons (Matikainen-Ankney et al., 2023). Moreover, blocking synaptic transmission from D1R-MSNs, but not D2R, reduces lever-pressing force during food seeking and attenuates HPF-induced weight gain (Matikainen-Ankney et al., 2023). However, despite recent evidence supporting the dichotomy between NAc D2R and D1R-containing neurons in driving motivation toward reinforcing stimuli such as drugs or food (Gerfen, 2023; Sandoval-Rodríguez et al., 2023; Swinford-Jackson et al., 2023), several studies indicated a non-canonical common role of NAc D1R and D2R in encoding positive valence/reward responses to drugs of abuse and food (Soares-Cunha et al., 2016; Gallo et al., 2018; Soares-Cunha et al., 2020; Joshi et al., 2021; Tan et al., 2022).

Glutamatergic plasticity in the NAc plays a key role in mediating the enhanced motivation for both food and drugs (Wolf and Tseng, 2012; Alonso-Caraballo et al., 2021). Imbalance in glutamatergic receptor expression is also a key feature of silent synapses, markers of synaptic reorganization. Recent studies have revealed that sucrose/junk food and cocaine increase the number of MSNs silent synapses in NAc (Alonso-Caraballo et al., 2021; Bijoch et al., 2023). Overall, these data suggest strong similarities between food- and drug-induced synaptic changes in NAc (Graziane et al., 2016; Terrier et al., 2016).

### 2.3. The ventral tegmental area

Every drug with abuse potential directly or indirectly acting on DA neurons in the ventral tegmental area (VTA) causes increased DA in the NAc (Volkow et al., 2019). In the context of eating behaviors, the hyperactive VTA DA-ergic projections lead to enhanced incentive salience or craving for food (Jerlhag et al., 2009). Food can act through the neural input from the taste buds and hormones released by the digestion and absorption of food (Alonso-Alonso et al., 2015). However, recent findings support the notion that direct stimulation of the gastrointestinal tract with nutrients or optical activation of gut-innervating vagal sensory neurons is sufficient to induce DA release in brain circuits controlling food intake. Interestingly, the changes in extracellular DA levels reflect the caloric load of the substance, even in the absence of taste receptor signaling (de Araujo et al., 2008; Han et al., 2018; Schatzker et al., 2020). VTA neurons express receptors for several peptides and hormones regulating homeostatic signals and influencing the responses to drugs. Among these, ghrelin has been reported to affect VTA DA-neuron firing rate and to increase the intake of HPF, the cocaine-induced locomotion, and conditioned place preference; by contrast, antagonism of ghrelin receptors reduces the development of nicotine and cocaine sensitization (Abizaid et al., 2006; Jerlhag et al., 2009; Skibicka et al., 2011; Wellman et al., 2011; Schuette et al., 2013; Cepko et al., 2014; Dunn et al., 2019).

Leptin, an adipose-derived hormone, modulates DA neurotransmission in the mesoaccumbens pathway acting on its receptors in VTA. This decreases the incentive value of both palatable food and substances of abuse such as cocaine and heroin (Figlewicz et al., 2003; Hommel et al., 2006; Morton et al., 2009; Shen et al., 2011; Meye and Adan, 2014; D’Cunha et al., 2020). Insulin inhibits dopaminergic VTA-NAc projections through the activation of the Akt–mTOR pathway and retrograde endocannabinoid signaling. This suppresses glutamate release and increases DA reuptake thereby upregulating DA transporter (DAT) and attenuating reward for HPF and drug (Iniguez et al., 2008; Bruijnzeel et al., 2011; Kenny, 2011; Mebel et al., 2012; Labouèbe et al., 2013; Tiedemann et al., 2017; Naef et al., 2019). In addition, signaling of peptides such as GLP-1 (involved in glucose regulation) and orexin (engaged in feeding behaviors) have been involved in reward regulation and dysfunctional responses toward food and drugs of abuse (Merchenthaler et al., 1999; Rinaman, 2010; Alhadeff et al., 2012; Erreger et al., 2012; Egecioglu et al., 2013; Graham et al., 2013; Shirazi et al., 2013; Engel and Jerlhag, 2014; Bentzley and Aston-Jones, 2015; Saad et al., 2019; Jamali et al., 2021). Overconsumption of HPFs can also induce dopaminergic adaptations within the VTA, such as a reduction of TH (both mRNA and protein levels), catechol-O-methyl transferase (DA degrading enzyme), and DAT, together with D1R and D2R expression (Vucetic et al., 2012; Carlin et al., 2013; Sharma and Fulton, 2013; Decarie-Spain et al., 2016).

### 2.4. The amygdala

The amygdala is primarily involved in memory, decision-making, and emotional response. As for NAc MSNs, stimulation or increased neuronal activity of D1R central amygdala (CeA) neurons enhances food seeking and is associated with incubation of drug seeking. Conversely, D2R stimulation suppresses food seeking and its reduced activity is associated with the incubation of drug craving (Kim et al., 2017; Venniro et al., 2017). Many of the long-term emotional disturbances associated with the withdrawal/negative stage of the addiction cycle have been related to dysfunctional activity of the CeA, which processes painful and pleasurable experiences (Roberto et al., 2017; Horseman and Meyer, 2019).

Negative emotional states and withdrawal symptoms are crucial factors of relapse. Incubation of drug seeking during abstinence has been observed in humans and animal models, and several studies outlined the involvement of the amygdala in mediating these behaviors (See et al., 2003; Lu et al., 2005; Smith and Aston-Jones, 2008; Li et al., 2015; Roura-Martínez et al., 2020; Pagano et al., 2023). Interestingly, blocking CB1 receptor signaling in the CeA can precipitate a negative emotional state in rats withdrawn from chronic intermittent access to HPF. This is similar to that seen in cannabinoid- and opiate-dependent subjects (Blasio et al., 2013), which suggests a link between compulsive eating and drug taking. Indeed, neural stimulation of CeA can strongly increase incentive motivation for natural and drug rewards (Tom et al., 2019; Warlow et al., 2020; Warlow and Berridge, 2021). It has been reported that kinase Cδ-expressing neurons mediate negative valence, satiation, and conditioned taste aversion, while prepronociceptin-expressing cells are suggested to be involved in assigning positive valence and enhanced motivation to HPF (Hardaway et al., 2019).

## 3. Modeling of food addiction in rodents

Modeling human psychiatric disorders in animals is challenging, particularly in the addiction field where the validity of rodent models has been argued to be restricted to “face” similarities (Hebebrand et al., 2014; Hebebrand and Gearhardt, 2021). This is even more evident in the FA research due to the debate on construct validity which leads to a non-univocal interpretation of the collected results (Salamone and Correa, 2013; Rogers, 2017; Sarkar et al., 2019; American Psychiatric Association, 2022). Nevertheless, literature so far collected in preclinical models suggests how specific foods (Berridge and Robinson, 2016; Wiss, 2022) can induce, under specific conditions, pathological manifestations accepted as valid measures of SUD symptoms (Deroche-Gamonet et al., 2004; Hone-Blanchet and Fecteau, 2014; Shriner and Gold, 2014). Along with the conceptual framework borrowed from SUDs, many of the behavioral tests used to investigate addiction-like eating behavior in rodents are modified tests of pathological drug use that use food as a primary reinforcer (Moore et al., 2019; Brown and James, 2023).

The earliest preclinical findings on food addiction-like behaviors derive from studies aimed primarily at the manipulation of the energy-homeostatic and metabolic aspects of feeding in the context of the study of obesity, which is not included among EDs but is clinically often associated with them (Berridge et al., 2010). Studies using prolonged free access (i.e., *ad libitum*) to HPFs have shown escalation in consumption beyond homeostatic needs (Valdivia et al., 2015; Kreisler et al., 2017; Wiss et al., 2018) that parallels escalation in drug addiction (Deroche-Gamonet et al., 2004; Johnson and Kenny, 2010). Similarly, free extended access to a palatable diet has been shown to induce behavioral and neurobiological alterations of tolerance induced by repeated or prolonged exposure to drugs of abuse (Ahmed et al., 2000; Dimitriou et al., 2000; Ahmed et al., 2002; Avena, 2010; la Fleur et al., 2011; Wojnicki et al., 2015; Parnarouskis and Gearhardt, 2022).

Continuous access to HPFs also produces an increase in body weight, complicating the distinction between overweight and overeating behavior (Corwin, 2006; Davis, 2013). Manipulations that provide alternating, intermittent exposures to HPFs aim to overcome this issue (Cottone et al., 2008; Emilien and Hollis, 2017; American Psychiatric Association, 2022). Intermittent or limited access to specific food rewards has been shown to promote a gradual escalation of preferred food intake across time, culminating in consumption of larger amounts during the first period in which food is available again (Avena et al., 2005; Cottone et al., 2008).

Intermittent access to substances/food produces more robust behavioral addiction-like manifestations than continuous access (Dimitriou et al., 2000; Kinzig et al., 2008; Corwin et al., 2011; Patrono et al., 2015; Wojnicki et al., 2015; Garcia et al., 2020; Vazquez-Herrera et al., 2021). Addiction-like increased consumption in the first period following re-exposure has been reported in rodents when they had intermittent period access shorter than 1 h daily (Corwin, 2004; Cottone et al., 2008; Giuliano et al., 2012; Halpern et al., 2013; Schulte et al., 2015; Wojnicki et al., 2015; Lee et al., 2020). This is similar to substance-induced behaviors in which more pronounced effects were observed with brief and limited access than with extended access (Kreisler et al., 2017; Spierling et al., 2020). Interestingly, “sporadic” exposure (2 h once weekly) to HPF in the absence of physiological stress induces a pathological phenotype after some weeks (Czyzyk et al., 2010), an observation also reported for nicotine (Miller et al., 2001) or ketamine (Trujillo et al., 2008).

Despite the difficulty in comparing results from these models due to diversities in the schedule of exposure (frequency and duration), intervening variables (e.g., concomitant slight food deprivation), and the type of test used, these models suggest a possible presence of incubation underlying the shift from a normal to a dysfunctional feeding behavior. In SUD models, this effect can be due to a hyper-evaluation of the palatable food when unavailable as well as devaluation of the less preferred alternative when a stable alternation has been acquired. Accordingly, food seeking in mice increases following prolonged abstinence from palatable food during self-administration training (Grimm et al., 2005; Krasnova et al., 2014; Darling et al., 2016; Madangopal et al., 2022). Uncertainty about the availability of the desired food may contribute to addiction-like behaviors by engaging in stress response (Cottone et al., 2009; Corwin et al., 2011). Interestingly, behavioral signs of a negative emotional state and increased stress responsivity are documented in animals that have access to high-fat food (Sharma et al., 2013), sucrose solutions (Galic and Persinger, 2002; Avena et al., 2005; Pickering et al., 2009; Yakovenko et al., 2011), or a combination of both (Teegarden and Bale, 2007) withdrawn, resembling the “deprivation effects” observed in models of intermittent drug and alcohol access (Rodd et al., 2004; Wang et al., 2004; George et al., 2007).

Abstinence from HPFs produces an anhedonic state and increased anxiety-like behaviors in rodents (Colantuoni et al., 2002; Yakovenko et al., 2011; Sharma et al., 2013; Ulrich-Lai et al., 2015; Spierling et al., 2020). Other reports also demonstrate an increase in responding to cues previously paired with high-fat foods, sucrose, and saccharin after abstinence (Aoyama et al., 2014; Darling et al., 2016; Dingess et al., 2017), parallel to response to drug-paired cues observed after abstinence (Epstein et al., 2016; Grimm, 2020).

The escalation in consumption has been proposed to be sustained by different mechanisms, not mutually exclusive, such as tolerance (as increased reward threshold) and opponent processes engagement (aversive state), suggesting that increased wanting is not sufficient to define the addiction (Cottone et al., 2009; Berridge and Robinson, 2016; Koob and Volkow, 2016; Parnarouskis and Gearhardt, 2022).

As for drugs (Volkow and Morales, 2015), the link with the stress system is further strengthened by evidence that exposure to environmental conditions, including shock (Hagan et al., 2002, 2003), isolation rearing (Blanco-Gandía et al., 2018), consume frustration (Cifani et al., 2009; Micioni Di Bonaventura et al., 2017; Anversa et al., 2020), forced swimming (Consoli et al., 2009), chronic variable stress (Pankevich et al., 2010; Thompson et al., 2015), and reduced maternal care early in life (Jahng, 2013) coupled with shock (Hancock et al., 2005) can powerfully influence the behavioral approach to palatable food. In turn, either continuous or intermittent palatable food intake blunts acute stress responses both in human and rodent studies (Pecoraro et al., 2004; Kinzig et al., 2008; Ulrich-Lai et al., 2015), supporting the hypothesis that palatable food has “comforting” effects that may promote its intake and relapse behaviors after abstinence (Avena et al., 2008; Cottone et al., 2009; Ulrich-Lai et al., 2010; Parylak et al., 2011).

Food restriction has been widely used in different models because it represents both an environmental source of stress able to influence addiction-like behaviors (Sinha and Jastreboff, 2013) and a condition mimicking certain aspects of diet regulation reported in humans, typically consisting of limitation of caloric intake (Stice and Burger, 2015). Boggiano’s model shows how cycles of energy restriction/refeeding (as with foot shock at the end of the final cycle) promote in rats more HPF consumption than foot shock or a history of restriction alone (Hagan et al., 2003; Chandler-Laney et al., 2007; Blanco-Gandía et al., 2018).

Many binging behavior models use modified versions of this protocol, with changes in the length of each component of the cycle, the type of binge food, the kind of acute stress administered, and the species of rodent (Hancock et al., 2005; Cifani et al., 2009; Consoli et al., 2009; Pankevich et al., 2010). In the model proposed by Hoebel, however, animals received palatable food during a period of mild food deprivation and showed an enhanced response to rewarding food as evidenced by the short-term (1 h) intake. The intake is higher than rats that received the palatable food only twice (Avena et al., 2008).

Evidence from rodent models also demonstrates the possibility to investigate eating despite negative consequences, a hallmark characteristic of SUD behaviors (Deroche-Gamonet et al., 2004; Vanderschuren and Everitt, 2004; Boggiano and Chandler, 2006; Everitt et al., 2008). Johnson and Kenny (2010) reported that rats with unrestricted access to a cafeteria diet continued to compulsively consume it despite the presence of an aversive conditioned stimulus (foot-shock-paired light), whereas rats previously fed with only regular chow and/or given restricted access to the high-fat/high-sugar diet significantly decreased their palatable food consumption in the presence of the aversive conditioned stimulus.

Oswald et al. (2011), Krasnova et al. (2014), and Rossetti et al. (2014) demonstrated how rats that developed binge-like intake after alternate HPF exposure persisted in self-administration despite the harmful consequences (foot shock). Similarly, rats withdrawing from intermittent access to a palatable diet compulsively sought and consumed a sugary diet while they were facing aversive conditions (the enlightened aversive compartment in a light/dark conflict box) (Calvez and Timofeeva, 2016). Work from our group has investigated the willingness to risk (shock-paired light presence) in order to consume a rewarding food after an alternated prolonged food exposure, evidencing in mice a critical role for genotype (Latagliata et al., 2010; Patrono et al., 2015).

## 4. Discussion

Differences and similarities between food and drug responses have sparked debate about whether FA could be a valid construct to define a clinical disorder. Due to the clinical evidence of the capability of HPF to promote specific features of SUDs, such as loss of control, craving, risky use, tolerance, and withdrawal, diagnostic tools aim to shed light on this issue. Moreover, preclinical studies modeling FA have attempted to investigate the interaction between biological and environmental factors in maladaptive behaviors toward drugs of abuse and food and have identified some common neurobiological substrates and alterations that could be coherently framed within the incentive salience theory of addiction. To clearly define FA as a disorder *per se*, however, including as a non-substance-related addiction, it is critical to understand how and why it emerges. The heterogeneity of results obtained by FA models should therefore be acknowledged and embraced, systematically investigating the role of specific variables on the development of the different behavioral features that compose drug addiction-like behaviors. Reaching these goals could lead to the development of specific and effective treatments for FA.

## Author contributions

AP, DM, and GC selected literature. AP, DM, GC, LB, RV, and MD wrote the manuscript. All the authors provide approval for publication of the content.

## Conflict of interest

The authors declare that the research was conducted in the absence of any commercial or financial relationships that could be construed as a potential conflict of interest.

## Publisher’s note

All claims expressed in this article are solely those of the authors and do not necessarily represent those of their affiliated organizations, or those of the publisher, the editors and the reviewers. Any product that may be evaluated in this article, or claim that may be made by its manufacturer, is not guaranteed or endorsed by the publisher.
